# Gut Microbiota Composition and Diversity in Different Commercial Swine Breeds in Early and Finishing Growth Stages

**DOI:** 10.3390/ani12131607

**Published:** 2022-06-22

**Authors:** Jianfeng Ma, Jingyun Chen, Mailin Gan, Lei Chen, Ye Zhao, Yan Zhu, Lili Niu, Shunhua Zhang, Li Zhu, Linyuan Shen

**Affiliations:** 1 Department of Animal Science, College of Animal Science and Technology, Sichuan Agricultural University, Chengdu 611130, China; 2020202051@stu.sicau.edu.cn (J.M.); 2020302110@stu.sicau.edu.cn (J.C.); ganmailin@stu.sicau.edu.cn (M.G.); chenlei815918@sicau.edu.cn (L.C.); zhye@sicau.edu.cn (Y.Z.); niulili@sicau.edu.cn (L.N.); 14081@sicau.edu.cn (S.Z.); 2 Farm Animal Genetic Resource Exploration and Innovation Key Laboratory of Sichuan Province, Sichuan Agricultural University, Chengdu 611130, China; 3 College of Life Science, China West Normal University, Nanchong 637009, China; zhuyan0720@stu.cwnu.cn

**Keywords:** growth curve, gut microbiota, 16S rRNA, swine, breed, production stage

## Abstract

**Simple Summary:**

The gut microbiota are involved in the metabolism of nutrients, and the growth and development of pig is strongly influenced by the gut microbiome. To maintain the integrity of the intestinal barrier and promote the digestion and absorption of nutrients and other physiological activities, it is beneficial if the host has a stable gut microbial community. The composition of the gut microbiota is influenced by many factors, such as genetic and environmental factors, and it changes with age. Throughout pig growth and development, the porcine gut microbiota constantly changes in composition. This study investigated the regulation of growth and development of body weight and body size index. We further examined changes in gut microbiota during early and finishing growth stages in Duroc, Landrace and Yorkshire pigs. Results showed that the microbiota of Landrace and Yorkshire pigs were more similar compared with Duroc pigs. There were significant differences in gut microbiota in the early and late growth stages. This study underlines the longitudinal variation in breed and lateral variation in age in gut microbiota.

**Abstract:**

The gut microbiota affects the metabolism, health and growth rate of pigs. Understanding the characteristics of gut microbiota of different pig breeds at each growth stage will enable the design of individualized feeding strategies. The present study aimed to compare the growth curves and development patterns of pigs of three different breeds (Duroc, Landrace and Yorkshire) using the mathematical models Gompertz, Logistic, Von Bertalanffy and Richards. For Duroc pigs, the Gompertz model showed the highest prediction accuracy (R^2^ = 0.9974). In contrast, the best models for Landrace and Yorkshire pigs were Richards (R^2^ = 0.9986) and Von Bertalanffy (R^2^ = 0.9977), respectively. Path analysis showed that body length (path coefficient  =  0.507) and chest circumference (path coefficient  =  0.532) contributed more significantly to the body weight of pigs at the early growth stage, while hip circumference (path coefficient  =  0.312) had a greater influence on pig body weight in the late growth stage. Moreover, the composition of the gut microbiota of pigs at two growth stages (60 kg of body weight in the early growth stage and 120 kg in the finishing stage) was studied using 16S rRNA sequencing technology. Variations in gut microbiota composition of pigs at different growth stages were observed. KEGG pathway enrichment analysis of annotated metagenomes revealed that protein synthesis and amino acid metabolism pathways were significantly enriched in pigs at the early growth stage, which may be related to nutritional requirements of pigs during this stage. This study confirmed longitudinal variation in the gut microbiota of pigs pertaining to age as well as lateral variation related to pig breed. The present findings expand the current understanding of the variations in swine gut microbiota during production stages.

## 1. Introduction

Mathematical models have been widely used to determine the growth curve of animals [1]. The growth curve of animals reflects the interaction pattern between hereditary and environmental factors, and usually has an S shape with inflection points and asymptotes [2,3]. In this context, modeling the growth curve of pigs has several benefits, e.g., for exploring growth differences between different breeds, determining nutritional needs of pigs in different production stages, easily predicting the weight of older pigs and for predicting slaughter weight to maximize breeding profit [4]. Thus, establishing nonlinear mathematical models has become a main research method. The most frequently used animal growth curve models include Gompertz, Logistic, Von Bertalanffy, Bridges, Richard and others. While many studies have described growth curve models for different pig breeds, no accepted best model has been identified. Therefore, establishing growth curves for different swine breeds and feeding environments will help animal breeders to understand pig herd developmental status, hence offering opportunities to design strategies for adjustment.

The gut microbiota is essential in mammalian health and disease [5]. The interaction between gut microorganisms and the host occurs throughout life [6]. The composition of gut microbiota affects overall pig physiology, including feed conversion ratio [7,8]. With the rapid development of high-throughput sequencing technology, research on the gut microbiota composition of domestic animals has advanced extensively. It has been shown that the gut microbiota of pigs fluctuates dynamically throughout developmental stages. Thus, understanding alterations in the pig gut microbiota related to growth stages is required to design a rational feeding strategy.

Pig gut microbiota are mainly comprised of anaerobic and facultative anaerobic bacteria, among which representatives of the phyla Firmicutes and Bacteroidetes correspond to more than 90% of the pig gut microbiota population. The gut microbiota impact pig metabolism and health [9]; pig intestines are sterile during the embryonic period, but the vertical transmission of gut microorganisms occurs from mother to fetus in parturition, being dominated by bacterial species *Escherichia coli* and *Staphylococcus* spp. [10]. Despite their low abundance, these species may accelerate gut microbiota development in pigs during early developmental stages [11].

In addition, it has been shown that the diversity of pig gut microbiota increases with age [12]. Moreover, around the growth curve inflection point, pigs have an active metabolism, and feed utilization efficiency may be affected by gut microbiota composition. During lactation, Lactobacillus and Streptococcus are the main dominant genera. From lactation to weaning, the number of endogenous lactic acid bacteria and anaerobic bacteria decreases, while the number of exogenous pathogenic bacteria, such as E. coli and Salmonella, increases [13], which is related to diarrhea in piglets. In particular, weaning causes dramatic changes in piglet gut microbiota, which are particularly illustrated by fluctuations in the relative abundance of the genus Prevotella; prior to weaning, the relative abundance of Prevotella in piglets is 6.66%, whereas after weaning it initially decreases and then increases, reaching 22.70% on day 70 of age [14,15]. The gut microbiota community gradually stabilizes in nursery piglets. Feed nutritional composition becomes the main factor influencing gut microbiota composition at this stage. Latest research has shown that variation in the expression of the ABO gene was related to decreased abundance of Erysipelotrichaceae by reducing GalNAc levels in the gut. In addition, it has been demonstrated that genetic factors can alter gut microbiota composition [16]. Yorkshire, Landrace and Duroc, three famous commercial pig breeds, are widely used in the pig industry. Growth characteristics of different pig breeds exhibit differences because of distinct genetic selection processes. Duroc pig is commonly the terminal male parent of the DLY pigs (Duroc × Landrace × Yorkshire) due to its high meat quality and large muscle mass. Therefore, it is worth exploring the influence of breed and genetic background on the pig gut microbiota composition.

Thus, the present study aimed to employ different mathematical models to establish growth curves of different pig breeds from birth to finishing developmental stages (0–180 days of age). Additionally, 16S rRNA gene sequencing to explore the differences in gut microflora composition and functional diversity between different commercial swine breeds during grower (approximate body weight = 60 kg) and finishing (approximate body weight = 120 kg) stages. The results discussed herein broaden the current understanding of gut microbiota variability across pig production stages, thus providing the basis for developing strategies to improve pig health and production performance.

## 2. Materials and Methods

### 2.1. Ethics Statement

All experimental procedures detailed below were approved by the Animal Ethical and Welfare Committee of Sichuan Agricultural University, Chengdu, China (No. 2020202051).

### 2.2. Animals and Sample Collection

Animals used in the experiments described herein were provided by a pig breeding company in Sichuan Province, China. All animals had free access to feed and water, and were housed under similar environmental conditions with ambient temperature around 25–35 °C. The levels of crude protein, trace minerals, vitamins and energy in the diet met or exceeded the recommendations of the National Research Council (NRC, 2012) for different production stages. In total, 6379 body weight measurements of gilts from birth to day 180 of age were applied for fitting growth curves, namely, Duroc (*n* = 505), Landrace (*n* = 2120) and Yorkshire (*n* = 3754). Fresh fecal samples were collected from individual pigs in grower (average body weight = 63.23 ± 5.49 kg) and finishing (average body weight = 123.5 ± 8.25 kg) stages: Duroc at grower (DG, *n* = 5); Landrace at grower (LG, *n* = 5); Yorkshire at grower (YG, *n* = 5); Duroc at finishing (DF, *n* = 5); Landrace at finishing (LF, *n* = 5); Yorkshire at finishing (YF, *n* = 5). Data on the average body weight of animals of each breed included in the study are given in Table S1. Each fecal sample was randomly collected from herd, and no longer than one minute from excretion to collection to ensure sample freshness. Fecal samples were stored in 2 mL centrifuge tubes and transported in an ice bath. All samples were stored at −80 °C in an ultrafreezer until DNA extraction.

### 2.3. Growth Curve Models

A total of four different growth curve models were used in the present study, namely three three-parameter models (Gompertz [17], Logistic [18] and Von Bertalanffy [19]) and a four-parameter Richards [19,20] model (Table 1). Parameters such as R^2^, Akaike’s information criterion (AIC), and root mean square error (RMSE) were determined and used to compare fitted models. R^2^ is the degree of fitting, calculated by the equation of R^2^ = 1 − RSE/RST, in which RST was the sum of squares of deviations and RSE represents the residual sum of squares. AIC was calculated as ‘−2log-likelihood + 2K’, where log-likelihood is the maximum likelihood, K is the number of parameters in the model and N is the sample size. GraphPad Prism software v.8.0 was used to estimate the model parameters A, B, K, m and comparative indicators.

### 2.4. Pearson Correlation and Path Analyses

To investigate the regularity of body size indexes and body weight (BW) at different developmental stages, we measured 270 pigs’ body sizes, i.e., a sample pool including Duroc, Landrace, Yorkshire. These measurements were divided into early (10–60 kg; *n* = 147) and late (60–120 kg; *n* = 123) growth stages. Differences between the two growth stages were compared, but the genetic background was not considered. Body size indexes included body length (BL), body height (BH), chest circumference (CC), abdominal circumference (AC), hip circumference (HC), chest width (CW), chest depth (CD) and cannon bone circumference (CBC). Pearson correlation and path analysis were performed using SPSS software (v.26.0; SPSS Inc., Chicago, IL, USA). Path analysis enables the study of direct and indirect effects simultaneously with multiple independent and dependent variables; thus, path analysis was used to partition the relative contributions of body size indexes using standardized partial-regression coefficients.

### 2.5. DNA Isolation and 16S rRNA Gene Sequencing

Total genomic DNA was extracted using the CTAB/SDS method [21]. DNA concentration and purity were verified by gel electrophoresis in 1% agarose gel. An aliquot of the sample was placed into a centrifuge tube and diluted to 1 ng/μL with sterile water.

PCR amplifications were performed using the diluted genomic DNA as a template. The hypervariable V3-V4 region of the 16S rRNA gene was amplified using the primer pair 515F-806R [22]. PCR amplification was performed using Phusion^®^ High-Fidelity PCR Master Mix (New England Biolabs, Ipswich, MA, USA) following the manufacturer’s instructions. PCR products were purified by agarose gel electrophoresis on 2% agarose gels. PCR products of between 400–450 bp were selected for PCR product purification using GeneJET (Thermo Scientific, Waltham, MA, USA) following the manufacturer’s instructions.

### 2.6. Data Analysis

Sequencing data analysis was conducted as previously reported [23]. Briefly, paired-end reads were merged using FLASH v.1.2.7 (http://ccb.jhu.edu/software/FLASH/, accessed on 22 January 2022). Quality control on raw tags for cleaning data was performed using QIIME v.1.7.0 (http://qiime.org/index.html, accessed on 22 January 2022). Tags were compared with the reference database (Gold database, http://drive5.com/uchime/uchime_download.html, accessed on 25 January 2022) using the UCHIME algorithm [24]. Sequences with ≥97% similarity were assigned to the same operational taxonomic units (OTUs) using Uparse software v.7.0.1001 (http://drive5.com/uparse/, accessed on 25 January 2022). Representative sequences for each OTU was selected and the RDP classifier (http://sourceforge.net/projects/rdp-classifier/, accessed on 27 January 2022) was used [25] to annotate taxonomic information for each representative sequence. Sample sequence number was used with the least number of sequences for normalization. Subsequent analysis was based on normalized OTUs (Table S4).

Alpha-diversity metrics were calculated using QIIME v.1.7.0, which included observed species, Chao1, Shannon, Simpson, ACE and Good’s coverage indexes; observed species index measures the number of species per sample; Chao1 and ACE indexes estimate species richness; Good’s coverage index measures sequencing depth; Shannon and Simpson indicate species distribution diversity and evenness. PD whole tree index was calculated based on evolutionary distance, thus reflecting the affinities between species within the community. QIIME v.1.7.0 was used to calculate beta diversity based on unweighted UniFrac distance using principal coordinates analysis (PCoA). PCoA analysis was performed with the packages WGCNA, stat and ggplot2 in R software v.2.15.3. Phylogenies among identified OTUs were calculated by the unweighted pair-group method using arithmetic averages (UPGMA) clustering. Linear discriminant analysis (LDA) effect size (LEfSe) and T-test statistical analysis were used to determine significant differences in abundance among different sample groups [26]. LEfSe analysis was conducted using the LDA score of 3. OTUs functions were annotated against the Greengenes database using PICRUST [27]. Statistical differences in the Kyoto Encyclopedia of Genes and Genomes (KEGG) pathways were visualized using the STAMP software package [28].

## 3. Results

### 3.1. Comparison of Growth Curve Models

**Figure 1:**
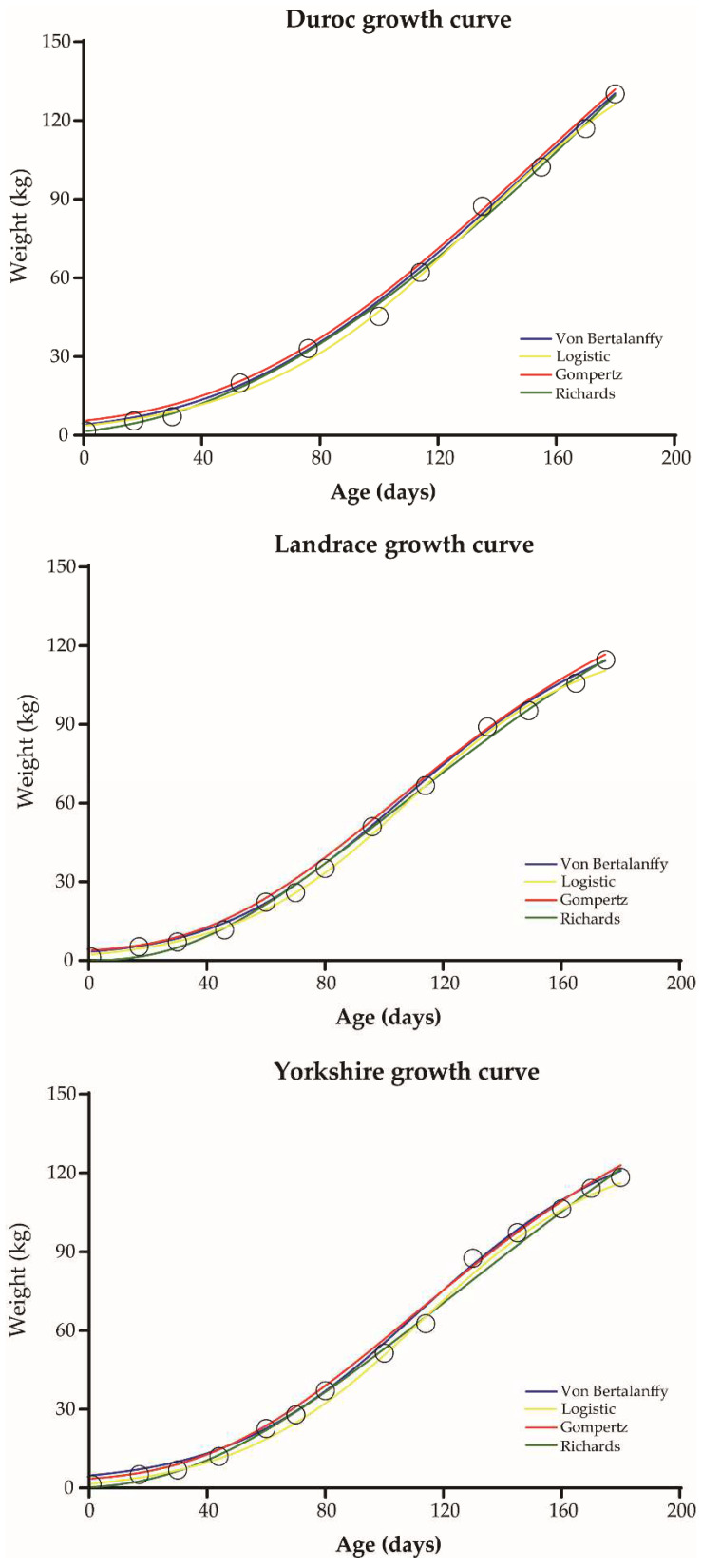
Fitted growth curves for Duroc, Landrace and Yorkshire pigs. The circle represents observed body weight. Lines in different colors indicate different growth curve models.

To compare growth differences of commercial pigs, four nonlinear models were used to fit the growth curves of three commercial pigs from nursery to finish (0–180 days of age), and the prediction accuracy of the models was compared (Table 1 and Table S2). Estimates of parameters pertaining to the four growth curve models are presented in Table 2. All four models fit a typical sigmoidal curve, and all fitted curves were close to the observed values (Figure 1). For Duroc pigs, the Gompertz model had the highest prediction accuracy (R^2^ = 0.9974). However, the optimal model for Landrace and Yorkshire pigs was Richards (R^2^ = 0.9986) and Von Bertalanffy (R^2^ = 0.9977), respectively (Table S2). In the optimal growth model, the growth rate for Duroc pigs peaked at 154.2 days, at which time body weight was 102.60 kg (Table 2). The growth inflection point for Landrace pigs was 104.9 days and 58.71 kg of body weight, but for Yorkshire pigs it was 123.1 days and 73.27 kg of body weight.

### 3.2. Correlation and Path Analyses of Body Weight and Body Size Indexes

**Figure 2:**
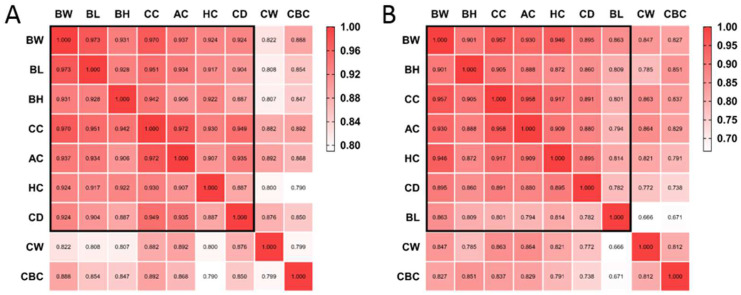
Heatmap of Pearson correlation coefficient matrix. (**A**) Early growth stage (10–60 kg of body weight); (**B**) late growth stage (60–120 kg of body weight). BW—body weight; BL—body length; BH—body height; CC—chest circumference; AC—abdominal circumference, HC—hip circumference; CW—chest width; CD—chest depth; CBC—cannon bone circumference.

Based on growth curves, two distinct patterns were exhibited in early and late growth stages. Pearson correlation analysis was conducted based on body size indexes and body weight in the early and late growth stages. Figure 2 depicts Pearson correlation coefficients between indicators; correlation coefficients with BW, BL, BH, CC, AC, HC and CD were higher both in early and late growth stages, whereas coefficients with CW and CBC were lower compared to other indicators.

Path analysis was conducted to explore the direct and indirect effects of body size indexes on the body weight of pigs at early and late growth stages. Both direct and total effects (the sum of direct and indirect effects, D + I) of BL (D: 0.507; D + I: 0.9728) and CC (D: 0.532; D + I: 0.9695) on body weight were higher in the early growth stage (Table 3). The total effects of indexes such as HC and CBC were low (Table 3). The direct and total effects of HC (D: 0.312; D + I: 0.9465) and CC (D: 0.454; D + I: 0.9569) were higher during the late growth stage (Table 3). The direct (from 0.507 to 0.203) and total effects (from 0.9728 to 0.8632) of BL on body weight decreased in the late growth stage (Table 4). According to the growth curve and path analysis of body size indexes, gilts at grower (63.23 ± 5.49 kg of body weight) and finishing (123.5 ± 8.25 kg of body weight) were selected for 16S rRNA gene sequencing for gut microbiome analysis, and differences in gut microbial composition and functions were compared.

### 3.3. Taxonomy and Diversity of Gut Microbiota of Gilts

**Figure 3:**
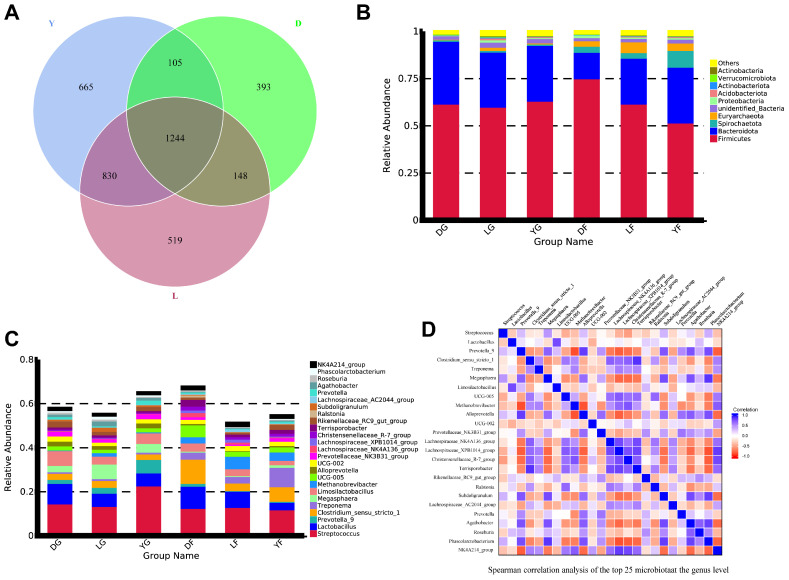
Number of operational taxonomic units (OTUs) and abundance analysis of annotated species in the gut microbiota of gilts of three different breeds at early and finishing growth stages. (**A**) Venn diagram of the number of OTUs in different pig breeds. D—Duroc; L—Landrace; Y—Yorkshire. (**B**) Histogram of the phylum-level relative abundance at the phylum level in different sample groups. DG—Duroc at early growth stage; LG—Landrace at early growth stage; YG—Yorkshire at early growth stage; DF—Duroc at finishing stage; LF—Landrace at finishing stage; YF—Yorkshire at finishing stage. (**C**) Histogram of the genus-level relative abundance in different sample groups. (**D**) Heatmap of Spearman correlation coefficients at the genus level.

After quality control, 48,286 valid OTUs were generated for each sample for subsequent analysis, which was clustered at 97% similarity. Alpha diversity analysis is shown in Table 5. Good’s coverage index was greater than 0.99 in all groups. Based on the dilution curve and rank abundance curve (Figure S1), sequencing depth was representative of the gut microbiome of gilts and could therefore be used in further analysis. Subsequently, shared and unique OTUs were analyzed among different pig breeds. Figure 3A shows that 1244 OTUs were shared among the three pig breeds. Moreover, 393 OTUs were uniquely found in Duroc pigs, 519 in Landrace pigs and 665 in Yorkshire pigs. The complete composition of annotated OTUs is shown in Table S3. In all groups, Firmicutes, Bacteroidota, Spirochaetota, Euryarchaeota and Proteobacteria were the main five phyla (over 95% of relative abundance) (Figure 3B). Firmicutes and Bacteroidota were the two most abundant phyla at both growth stages. However, the abundance of other phyla (Spirochaetota and Euryarchaeota) increased from growth to the finish stage (Figure 3B). At the genus level, Streptococcus was the most abundant, whereas the abundance of Lactobacillus varied among groups (9.33%, 5.96% and 5.89% in DG, LG and YG, respectively; 10.05%, 7.34% and 3.36% in DF, LF, and YF, respectively) (Figure 3C). In addition, the abundance of Megasphaera in pigs during the early growth stage was higher than in pigs at the finishing stage, whereas the relative abundance of Clostridium_sensu_stricto_1 showed an opposite trend (Figure 3C). Figure 3D shows the correlation between the top 25 most abundant genera in the gut microbiota of gilts at different growth stages based on Spearman’s correlation analysis.

### 3.4. Differences in Gut Microbiota Composition among the Three Pig Breeds

**Figure 4:**
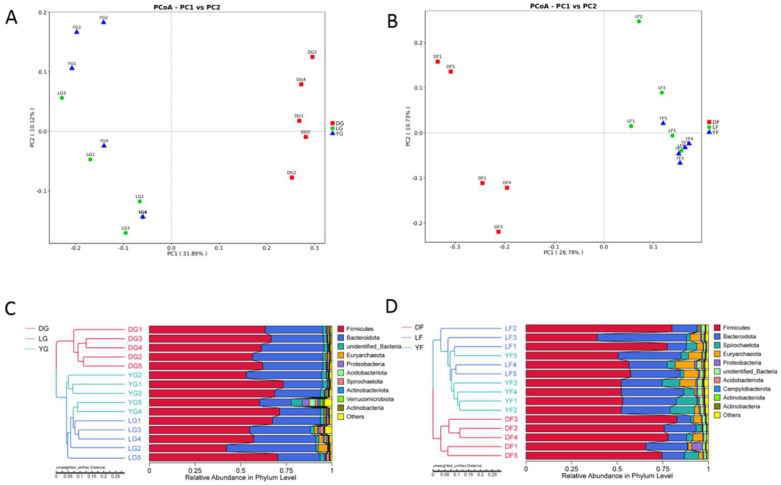
(**A**,**B**) Principal coordinate analysis (PCoA) and (**C**,**D**) unweighted pair group method with arithmetic means analysis (UPGMA) based on unweighted Unifrac distances of the phylum-level relative abundance in the gut microbiota of Duroc, Landrace and Yorkshire pigs at both early and finishing growth stages. DG—Duroc at early growth stage; LG—Landrace at early growth stage; YG—Yorkshire at early growth stage; DF—Duroc at finishing stage; LF—Landrace at finishing stage; YF—Yorkshire at finishing stage.

The gut microbiota composition in the three breeds included in the study was investigated further. PCoA analysis of unweighted Unifrac distances revealed differences in the gut microbiota composition of Duroc, Landrace and Yorkshire pigs at both early and finishing growth stages (Figure 4A,B). Clustering trees were built using UPGMA clustering based on unweighted Unifrac distances matrices (Figure 4C,D), which showed that the composition of the gut microbiota of the three pig breeds clustered separately. Interestingly, in both PCoA and UPGMA analysis, the gut microbiota composition of Landrace and Yorkshire pigs was more closely associated compared with the gut microbiota composition of Duroc pigs.

**Figure 5:**
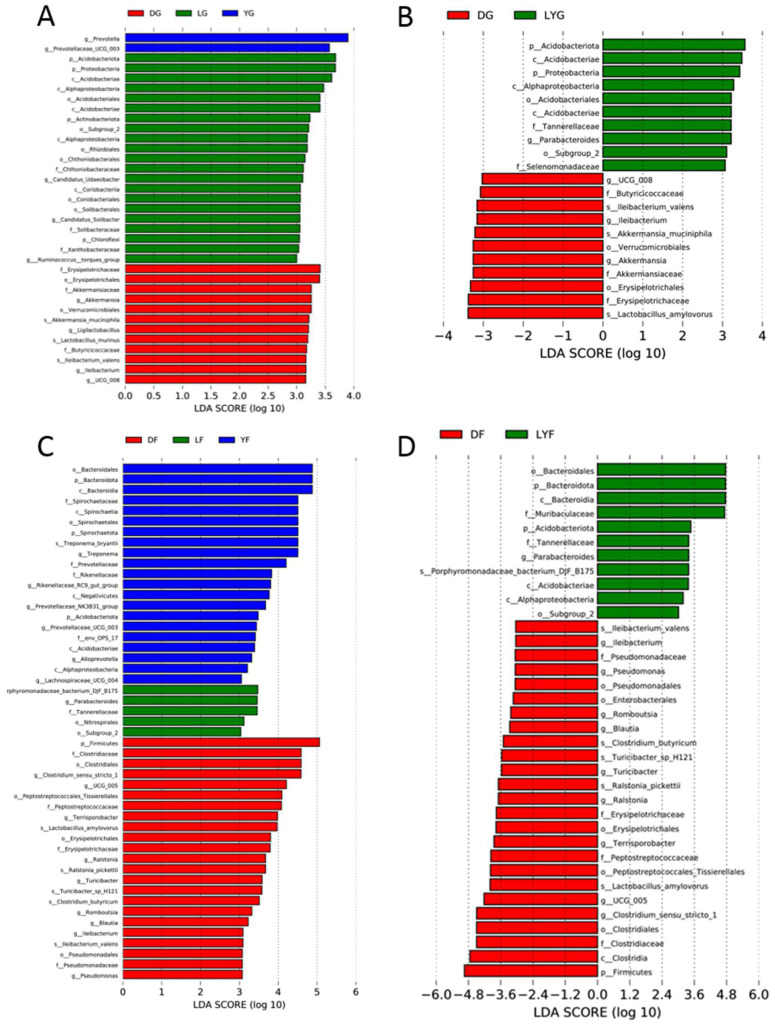
Differences in the gut microbiota composition of different pig breeds at early and finishing growth stages based on Linear discriminant analysis effect size (LEfSe). (**A**) Differential microbial species in the gut microbiota of Duroc, Landrace and Yorkshire pigs at the early growth stage. (**B**) Differential microbial species in the gut microbiota of Duroc, Landrace and Yorkshire pigs at the finishing stage. (**C**) Comparison between the gut microbiota composition in pigs of Duroc and the Landrace and Yorkshire (LY) breeds at the early growth stage. (**D**) Comparison between the gut microbiota composition in pigs of Duroc and LY at the finishing stage.

Additionally, LEfSe analysis was performed to determine the abundance of specific microbial taxa among the three pig breeds. A LDA score (log_10_) greater than three was considered the threshold. A total of 35 potential biomarkers were found in pigs at the early growth stage (2 in the YG group; 21 in the LG group; 12 in the DG group) and 49 at the finishing stage (21 in the YF group; 5 in the LG group; 23 in the DG group) (Figure 5). At the early growth stage, *Prevotella* and *Prevotellaceae_UCG_003* were the most abundant genera in the gut microbiota of Yorkshire pigs; *Acidobacteriota*, *Proteobacteria* and *Actinobacteria* were the most abundant genera in Landrace pigs; *Erysipelotrichaceae*, *Akkermansiaceae*, *Ligilactobacillus* and *Ileibacterium* were the most abundant genera in Duroc pigs (Figure 5A). At the finishing stage, the number of differential microbial taxa identified increased. *Bacteroidales*, *Spirochaetaceae*, *Treponema*, *Prevotellaceae_UCG_003* and *Alloprevotella* were the most abundant genera in the gut microbiota of Yorkshire pigs; *Tannerellaceae*, *Nitrospirales* and *Parabacteroides* were the most abundant in Landrace pigs; *Terrisporobacter*, *Tuicibacter*, *Blautia*, *Romboutsia* and *Ileibacterium* were the most abundant in Duroc pigs (Figure 5B). Interestingly, at both growth stages, the abundance of *Erysipelotrichaceae* or *Ileibacterium* in Duroc pigs differed substantially from those in the other two pig breeds (Figure 5A,B). Previously discussed PCoA and UPGMA clustering results indicated that the gut microbiota composition in Duroc pigs differed remarkably from that of the other two pig breeds (Figure 4). Therefore, Landrace and Yorkshire breeds formed a new group (LYG and LYF) for LEfSe analysis (Figure 5C,D). *Erysipelotrichaceae* could be a potential marker to distinguish the gut microbiota composition among the three pig breeds investigated in the present study. Moreover, the results of the unpaired *t*-test and metastats analysis validated the LEfSe data (Figure S2A,B). Finally, a cladogram was constructed to illustrate the phylogenetic differences in the gut microbiota composition among the three pig breeds investigated in the current study (Figure S2C).

### 3.5. Comparison of the Functions of the Gut Microbiota of Pigs at Early and Finishing Growth Stages

**Figure 6:**
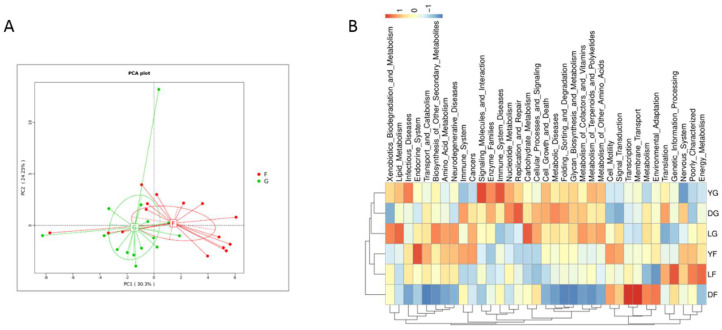
Predicted function of the gut microbiota of pigs of three different breeds at early and finishing growth stages based on KEGG pathway analysis using PICRUSt. (**A**) Principal component analysis of KEGG pathway abundance. G—early growth stage; F—finishing growth stage. (**B**) Clustering heatmap of KEGG pathway abundance in different sample group. DG—Duroc at early growth stage; LG—Landrace at early growth stage; YG—Yorkshire at early growth stage; DF—Duroc at finishing stage; LF—Landrace at finishing stage; YF—Yorkshire at finishing stage.

Significant differences were found in the composition of the gut microbiota of pigs of three different breeds at early and finishing growth stages. Therefore, metagenome functions were predicted using PICRUSt based on KEGG pathways. Predictive function richness was used to generate a principal component analysis (PCA) plot. Samples at the early and finishing growth stages were clustered separately (Figure 6A). Figure 6B shows the clustering heatmap of the predicted function of the gut microbiota in sample groups; samples of the early growth stage group (DG/LG/YG) were first clustered in one branch and then with samples of the finishing growth stage group, thus indicating that predicted functions of the gut microbiota of pigs of different breeds diverged between the two growth stages.

**Figure 7:**
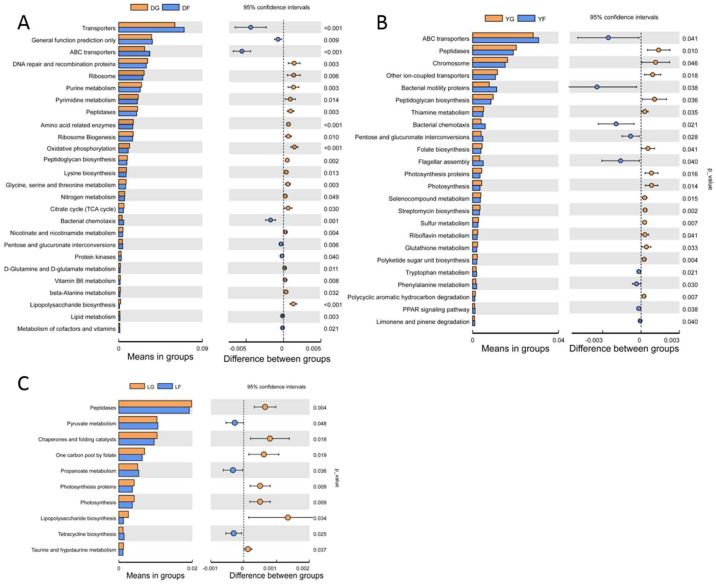
Differences in the abundance of annotated KEGG levels (level 3) of gut microbiota composition of pigs of three different breeds at the different growth stages based on T-test analysis. (**A**) Duroc pigs. (**B**) Landrace pigs. (**C**) Yorkshire pigs. Extended error bar plot significant differences between early and finishing growth stages with corrected *p* values shown on the right.

Then, a T-test analysis was carried out on the abundance of annotated KEGG levels (level 3) of gut microbiota composition of pigs of three different breeds at the different growth stages. The following pathways were significantly enriched in Duroc pigs when comparing the two growth stages: peptidases; amino-acid-related enzymes; ribosome biogenesis; lysine biosynthesis; glycine, serine and threonine metabolism; D-glutamine and D-glutamate metabolism (Figure 7A). In contrast, the most significantly enriched KEGG pathways in Yorkshire pigs at both growth stages were ABC transporters, peptidases, peptidoglycan biosynthesis, glutathione metabolism and tryptophan metabolism (Figure 7B). However, only a small number of KEGG pathways were differentially enriched in Landrace pigs at both growth stages, i.e., peptidases, pyruvate metabolism, propanoate metabolism and taurine and hypotaurine metabolism (Figure 7C).

## 4. Discussion

Four mathematical models were used in the present study to determine growth curves for predicting pig production performance. All models showed good fit, although other functions might be suitable for growth curve fitting [29]. Fit accuracy was different based on different models for the three pig breeds evaluated in the study. The best model for Duroc pigs was Gompert, whereas the best models for Landrace and Yorkshire pigs were Richards and Von Bertalanffy, respectively. This difference may be related to their intrinsic growth potential. Duroc pigs showed the largest inflection point related to age and body weight (154.2 days and 102.60 kg), while Landrace and Yorkshire pigs were relatively smaller. Previous studies reported a growth inflection point related to the body weight of Finnish Yorkshire pigs at 73.9 kg [30] and 71.9 kg [31], which was similar to the results of the present study (73.27 kg). Dragutin [3] et al. reported that the growth inflection point of DLY gilts (Yorkshire × Landrace sows and Duroc boars) was 121.04 days and 70.70 kg of body weight. However, Strathe [32] et al. showed that the inflection point of DLY gilts was 96 kg. In addition to genetic background, the diet and model accuracy may cause differences in estimated growth inflection point related to body weight.

Moreover, the relationship between body size and body weight were investigated between pigs at early and finishing growth stages. BL and CC had a greater effect on body weight at the early growth stage, while the contribution of HC and CC was greater at the finishing growth stage. These findings might be related to the earlier development of bones compared to muscle and adipose tissue in pigs. Thus, BL is likely associated with bone development, while HC can be mainly associated with meat production.

An interplay between the host and gut microbiota occurs throughout the animals’ lifetime. Gut microbial composition varies between individuals and affects health [33]. Over the past decade, many studies have investigated the influence of breed [8,34], age [35] and sex [36] on the variation of pig gut microbiota, and recent studies have greatly expanded current knowledge of the impact of host genes on the pig gut microbiota [16]. Throughout pig growth development, maximum growth rate is estimated by the growth inflection point, which suggests that metabolism and digestive functions are active. 

In the present study, the gut microbiota composition and its predicted functions in pigs of three different breeds were determined at early and finishing growth stages to describe longitudinal variation based on age and lateral variation based on breed. Firstly, we explored the diversity of the gut microbiota across breeds and growth stages. A different number of OTUs were identified in different pig breeds. The number of OTUs in the gut microbiota of Yorkshire pigs (YG = 1078.8 ± 114.87 and YF = 1181.2 ± 116.54) was higher than that in Landrace (LG = 1057.8 ± 208.06 and LF = 1080.6 ± 158.33) and Duroc pigs (DG = 934.0 ± 26.54 and DF = 886.8 ± 51.14); these findings were consistent with those reported by Pajarillo et al. [37] Collectively, it was observed that diversity in the gut microbiota of Landrace and Yorkshire pigs increased with age, as demonstrated by increased observed species, Shannon, Chao1 and Ace indexes; in contrast, diversity in the gut microbiota of Duroc pigs decreased, as demonstrated by decreased Chao1 and Ace indexes. Previous studies have shown that diversity in the gut microbiota increases with age in pigs [11]. In fact, gut bacterial diversity in 150-day-old pigs was higher than in early growing pigs [38]. However, Han et al. reported contrasting results; the gut microbiota diversity in finishing pigs (147 days old) was significantly lower than that in pigs in the growing stage (10–93 days old) [35]. As shown herein, the increase in diversity in the gut microbiota differed among the three pig breeds evaluated in the study. Firmicutes and Bacteroidetes were the two dominant phyla in the gut microbiota of pigs, which accounted for 80–90% of relative abundance, as described in several other studies [11,39,40]. The current study also revealed that the proportion of Firmicutes increased and that of Bacteroidetes decreased in Duroc and Landrace pigs with age. Similar results have been reported in humans and pigs [39]; however, the trend in Yorkshire pigs differed. This difference might be related to pig age, feed, environment and other factors. The composition of pigs’ diet depends on a commercial formulation and changes with the growth stage. Weaning is one of the most stressful events in a pig’s life, and their dietary patterns also change rapidly, leading to changes in gut microbiota. However, as pigs grow, their gut microbiota plateau [35]. Our study focused on the most rapid stage of pig growth (63.23 ± 5.49 kg) and the initial maturation stage (123.5 ± 8.25 kg). We observed the variability between the two stages, which may be because pigs are fed different diets in the different growth phases.

To explore variations in the gut microbiota among pig breeds, PCoA analysis was conducted based on unweighted UniFrac distances. Samples related to Landrace and Yorkshire pigs clustered together in PCoA plot and UPGAM cluster analysis; thus, the gut microbiota composition in these samples was comparable, as suggested previously [37]. Landrace and Yorkshire pig breeds are more genetically related compared to Duroc [41], which could explain the effect of breed on gut microbiota enrichment. Interestingly, the family *Erysipelotrichaceae* could be used as a marker to distinguish Duroc pigs from the other two breeds at both the early and finishing growth stages. A recent study showed that a 2.3 kb deletion in the N-acetyl-galactosamine transferase gene caused an altered abundance of *Erysipelotrichaceae* in pig gut microbiota, hence providing strong evidence for the influence of host genes on the abundance of specific bacteria in the gut [16]. Thus, it can be assumed that specific genetic backgrounds might affect microbial colonization as a result of altered levels of metabolites in the body. The higher abundance of *Erysipelotrichaceae* in the gut microbiota of Duroc pigs may be related to specific genes and can be considered a potential biomarker to distinguish these breeds; however, further experiments are needed to confirm this hypothesis. Previous studies showed that *Catenibacterium*, *Phascolarctobacterium* and *Subdoligranulum* were more abundant in Duroc compared with Yorkshire and Landrace [37]. *Catenibacterium* is a genus of Gram-positive and obligatory anaerobe bacteria that are mainly involved in the biosynthesis and metabolism of fatty acids. These observations may be due to breed-specific differences affecting gut function. A prior study found *Prevotella* and *Bacteroides* were higher in obese Gottingen pigs, while *Clostridium* was higher in lean Gottingen pigs. Another study suggested that Prevotella had a greater relative abundance in lean Ossabaw pigs, whereas Clostridium was richer in obese Ossabaw minipigs [42]. The specific structures or secreted products from these bacteria may alter the digestive, metabolic, endocrine and other functions of the pigs and eventually lead to differences in growth rate. Host genetics have an important influence on microbial diversity. In our study, we observed the similar gut microbiota composition of Yorkshire and Landrace. Previous studies have suggested that the microbial composition of Duroc, Landrace and Yorkshire breeds clustered together and differed from that of Chinese breeds such as Bama and Meishan sows [43]. Studies in mice have similarly shown that genetically similar mice have more similar bacterial compositions than genetically distant mice [44]. Differential composition of gut microbiota due to genetics was common in mammals. However, the mechanism behind this deserves further study.

Finally, KEGG pathway analyses were conducted based on PICRUSt to predict the functions of gut microbiota of pigs at different growth stages. In Duroc pigs at the early growth stage, the most enriched pathways were ribosome biogenesis, amino-acid-related enzymes, lysine biosynthesis, glycine and serine and threonine metabolism, which are involved in protein synthesis and amino acid utilization. Thus, these findings suggest that, compared with the gut microbiota of pigs at the finishing stage, the metabolic efficiency of gut microbes in pigs at the early growth stage may be higher to meet nutritional requirements and rapid growth rate. In contrast, bacterial chemotaxis and lipid metabolism were the most enriched pathways in Duroc pigs at the finishing stage. Fat deposition occurs in pigs mainly at the late finishing growth stages, and excessive body fat deposition may lead to chronic inflammation. Yang et al. identified numerous obesity-related bacteria in pig gut microbiota, including *E. coli* [45]. Moreover, pathways involved in protein synthesis (peptidases and peptidoglycan biosynthesis) were also found in Landrace and Yorkshire pigs. Given that differences in the function of gut microbiota observed herein were not similar among different pig breeds, it can be speculated that these might be related to differences in growth rates of evaluated pig breeds. For instance, the growth rate of Duroc pigs peaked on day 154, whereas that of Landrace and Yorkshire pigs peaked relatively early (on day 123.1 and 104.9, respectively). With age, gut microbiota composition and function in pigs changed. Thus, further research is necessary to determine whether changes are first produced by the gut microbiota and the host metabolism.

## 5. Conclusions

In the present study, different mathematical models were tested to predict the growth curves of pigs of three different breeds. Through path analysis, the correlation between body size and body weight were evaluated. In addition, the gut microbiota of pigs at the early and finishing growth stage was studied, and inter-breed differences were investigated. *Erysipelotrichaceae* was a potential biomarker to distinguish the growth performance of Duroc pigs compared to Landcaster and Yorkshire pigs, which was previously shown to be associated with the expression of the *ABO* gene. The present study shed a light on factors contributing to gut microbiota variability in production pigs, and the findings discussed herein provide the basis for further improving pig performance.

## Figures and Tables

**Table 1:** Growth curve models adopted in the study and related parameters.

Model	Equation	Parameters	Wi	Day at Inflection	Maximum Daily Gain
Logistic	$W_{t}=\frac{A}{1+Bexp\left(-\mathrm{Kt}\right)}$	A, B, K	A/2	(lnB)/K	$\frac{\mathrm{KWi}}{2}$
Gompertz	$W_{t}=\mathrm{Aexp}\left(-Bexp\left(-\mathrm{Kt}\right)\right)$	A, B, K	A/e	(lnB)/K	KWi
Von Bertalanffy	$W_{t}=A\;{\left(1-Bexp\left(-\mathrm{Kt}\right)\right)}^{3}$	A, B, K	8A/27	(ln3B)/K	$\frac{3\mathrm{KWi}}{2}$
Richards	$W_{t}=\frac{A}{{\left(1+Bexp\left(-\mathrm{Kt}\right)\right)}^{\frac{1}{m}}}$	A, B, K, m	$\frac{A}{{\left(m+1\right)}^{\frac{1}{m}}}$	$\frac{-\ln(m/B)}{K}$	$\frac{\mathrm{AK}}{2\left(m+2\right)}$

Note: W_t_—body weight in kg at the time t; t—age in days; A, B, K and m—specific parameters in the function; Wi—Weight of inflection; e—Euler number.

**Table 2:** Estimates of growth curve fitting parameters.

Model	Breed	A	B	K	m	Wi	Day at Inflection	Maximum Daily Gain
Logistic	D	163.2	34.12	0.02664	-	81.60	132.5	1.087
	L	123.3	37.93	0.03359	-	61.65	108.2	1.035
	Y	131.8	36.63	0.03195	-	65.90	112.7	1.053
Gompertz	D	278.9	4.690	0.01002	-	102.60	154.2	1.028
	L	156.3	5.277	0.01606	-	57.50	103.6	0.923
	Y	169.6	5.100	0.01500	-	62.39	108.6	0.936
Von Bertalanffy	D	573.1	0.8623	0.00440	-	169.81	216.1	1.120
	L	195.3	0.9574	0.01013	-	57.87	104.2	0.879
	Y	247.3	0.8958	0.00803	-	73.27	123.1	0.883
Richards	D	278.9	0.000868	0.01003	0.000185	102.61	154.1	0.699
	L	137.3	4.041	0.02268	0.3740	58.71	104.9	0.656
	Y	139.5	10.84	0.02568	0.6295	64.23	110.8	0.681

Note: D—Duroc; L—Landrace; Y—Yorkshire; A, B, K and m—specific parameters in the function; Wi—Weight of inflection.

**Table 3:** Path coefficients for direct and indirect effects of body size indexes on body weight in 10–60 kg stage.

Predictor Variables	Correlation	Direct Effects (D)	*p* Value	Indirect Effects (I)									Total Effects (D + I)
				BL	BH	CC	AC	HC	CW	CD	CBC	Total (I)	
BL	0.973	0.507	<0.01		−0.057	0.505	−0.169	0.081	−0.059	0.062	0.102	0.4656	0.9728
BH	0.931	−0.061	0.200	0.471		0.501	−0.164	0.081	−0.058	0.061	0.101	0.9923	0.9313
CC	0.970	0.532	<0.01	0.482	−0.057		−0.177	0.082	−0.064	0.065	0.107	0.4379	0.9695
AC	0.937	−0.182	<0.01	0.474	−0.055	0.517		0.080	−0.065	0.064	0.104	1.1188	0.9373
HC	0.924	0.088	0.045	0.465	−0.056	0.494	−0.165		−0.058	0.061	0.095	0.8361	0.9241
CW	0.822	−0.073	0.030	0.410	−0.049	0.469	−0.162	0.070		0.060	0.096	0.8944	0.8216
CD	0.924	0.069	0.139	0.459	−0.054	0.505	−0.170	0.078	−0.064		0.102	0.8553	0.9242
CBC	0.888	0.120	<0.01	0.433	−0.052	0.474	−0.158	0.069	−0.058	0.059		0.7678	0.8875

BL—body length; BH—body height; CC—chest circumference; AC—abdominal circumference; HC—hip circumference; CW—chest width; CD—chest depth; CBC—cannon bone circumference.

**Table 4:** Path coefficients for direct and indirect effects of body size indexes on body weight in 60–120 kg stage.

Predictor Variables	Correlation	Direct Effects (D)	*p* Value	Indirect Effects (I)									Total Effects (D + I)
				BL	BH	CC	AC	HC	CW	CD	CBC	Total (I)	
BL	0.863	0.203	<0.01		0.001	0.364	−0.058	0.254	0.040	0.026	0.034	0.660	0.8632
BH	0.901	0.001	0.983	0.1643		0.412	−0.065	0.272	0.047	0.028	0.044	0.900	0.9012
CC	0.957	0.454	<0.01	0.1628	0.001		−0.071	0.286	0.051	0.029	0.043	0.503	0.9569
AC	0.930	−0.074	0.302	0.1613	0.001	0.435		0.284	0.051	0.029	0.042	1.004	0.9299
HC	0.946	0.312	<0.01	0.1655	0.001	0.416	−0.067		0.049	0.029	0.040	0.635	0.9465
CW	0.847	0.060	0.152	0.1352	0.001	0.392	−0.064	0.256		0.025	0.042	0.787	0.8469
CD	0.895	0.033	0.496	0.1589	0.001	0.405	−0.065	0.279	0.046		0.038	0.863	0.8955
CBC	0.827	0.051	0.214	0.1363	0.001	0.380	−0.061	0.247	0.048	0.024		0.776	0.8266

BL—body length; BH—body height; CC—chest circumference; AC—abdominal circumference; HC—hip circumference; CW—chest width; CD—chest depth; CBC—cannon bone circumference.

**Table 5:** Alpha diversity of gut microbiome in pigs in early and late growth stages.

Groups	Observed Species	Shannon	Simpson	Chao1	Ace	Good’s Coverage	PD Whole Tree
DG	934.0 ± 26.54	6.12 ± 0.16	0.9393 ± 0.0070	1007.71 ± 30.95	988.36 ± 31.53	0.9977 ± 0.0001	71.5 ± 4.2
LG	1057.8 ± 208.06	6.11 ± 0.85	0.9232 ± 0.0484	1157.34 ± 201.06	1133.14 ± 200.7	0.9970 ± 0.0004	83.90 ± 13.38
YG	1078.8 ± 114.87	6.09 ± 0.38	0.9234 ± 0.0220	1195.22 ± 129.71	1163.51 ± 120.66	0.9966 ± 0.0003	86.90 ± 6.36
DF	886.8 ± 51.14	6.42 ± 0.25	0.9556 ± 0.0103	955.03 ± 58.56	937.04 ± 53.67	0.9979 ± 0.0001	78.00 ± 11.29
LF	1080.6 ± 158.33	5.97 ± 0.65	0.9114 ± 0.0500	1190.42 ± 181.05	1161.67 ± 172.1	0.9967 ± 0.0006	88.11 ± 10.83
YF	1181.2 ± 116.54	6.47 ± 0.51	0.9487 ± 0.0177	1302.62 ± 138.18	1266.83 ± 124.51	0.9965 ± 0.0003	90.87 ± 7.94

The observed species index shows the number of OTUs actually observed; Shannon and Simpson indices measure biodiversity; Chao1 and Ace indices reflect the microbial species richness; Good’s coverage index shows coverage of sequencing data; PD whole tree index reflects the diversity based on the phylogenetic tree; DG—Duroc at early growth stage; LG—Landrace at early growth stage; YG—Yorkshire at early growth stage; DF—Duroc at finishing stage; LF—Landrace at finishing stage; YF—Yorkshire at finishing stage. All values are reported as means ± standard deviation (SD).

## Data Availability

For the remaining data that may be relevant, the corresponding authors can be contacted.

## Supplementary Materials

The following supporting information can be downloaded at: https://www.mdpi.com/article/10.3390/ani12131607/s1, Figure S1: Dilution curve and rank abundance curve; Figure S2: Metastats analysis, unpaired *t*-test and cladogram from LEfSe analysis; (**A**) Heatmap of Metastats analysis; (**B**) The unpaired *t*-test of compositional abundance; (**C**) The cladogram of LEfSe analysis; Table S1: Body weight of pigs at different days of ages; Table S2: Comparison of goodness of fit in different models; Table S3: Species composition abundance at phylum and genus levels; Table S4: OTUs table.
