# Supplementary material for: Gut Microbiota Composition and Diversity in Different Commercial Swine Breeds in Early and Finishing Growth Stages
Source: Animals (Basel). 2022 Jun 22;12(13):1607. doi: 10.3390/ani12131607 (PMC9264831; doi:10.3390/ani12131607)
Supplement: Supplementary file 1 [file animals-12-01607-s001.zip › Tables S1-S2 & Figures S1-S2.pdf]

Table S1. Body weight of pigs at different days of ages

D			L			Y		
**Days of age**	**BW**	**Std**	**Days of age**	**BW**	**Std**	**Days of age**	**BW**	**Std**
1	1.645	0.314	1	1.321	0.284	1	1.362	0.293
17	5.475	0.894	17	5.282	0.777	17	5.134	0.717
30	7.176	1.246	30	7.086	1.385	30	6.902	1.505
53	20.000	1.631	46	11.720	1.844	44	12.046	1.009
76	33.128	6.819	60	22.281	3.110	60	22.690	3.149
100	45.375	1.576	70	25.889	4.509	70	27.892	4.541
114	62.150	3.377	80	35.119	6.074	80	37.139	4.444
135	87.333	2.853	96	51.000	2.000	100	51.422	4.825
150	98.182	9.595	114	66.727	6.433	114	62.667	1.434
170	116.972	9.992	135	89.050	3.873	130	87.605	2.826
180	130.167	4.337	149	95.208	8.231	145	97.353	9.324
			165	105.692	9.555	160	106.415	10.493
			175	114.609	12.369	170	114.082	11.410
			180	120.250	5.423	180	118.301	12.155

Note: D: Duroc; L: Landrace; Y: Yorkshire; BW: body weight; Std: standard deviation.

Table S2. Comparison of goodness of fit in different models

	D				L				Y			
	**Logistic**	**Gompertz**	**Von Bertalanffy**	**Richards**	**Logistic**	**Gompertz**	**Von Bertalanffy**	**Richards**	**Logistic**	**Gompertz**	**Von Bertalanffy**	**Richards**
R^2	0.9964	0.9974	0.9973	0.9974	0.9979	0.9982	0.9970	0.9986	0.9974	0.9968	0.9977	0.9975
AIC	36.49	32.91	33.24	40.24	28.24	26.25	33.02	28.63	33.95	36.47	31.74	38.28
RMSE	2.828	2.403	2.44	2.403	1.87	1.733	2.248	1.533	2.237	2.448	2.14	2.18

Note: D: Duroc; L: Landrace; Y: Yorkshire; R2: the degree of fitting; AIC: Akaike's information criterion; RMSE: root mean square error.

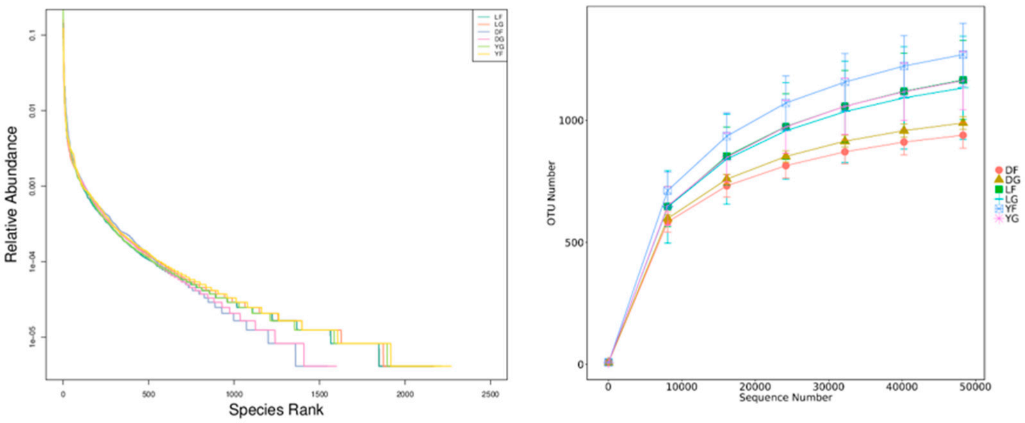

Figure S1. Dilution curve and rank abundance curve

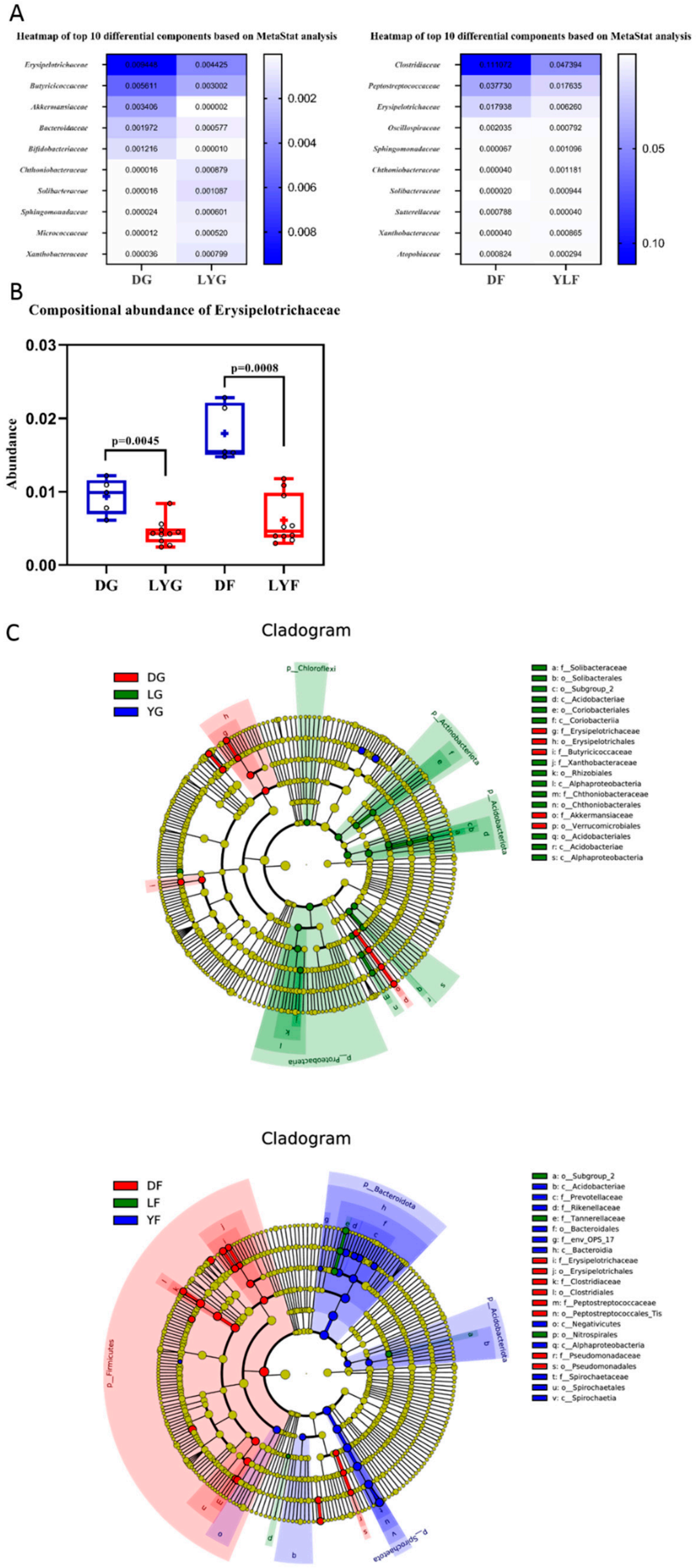

Figure S2. Metastats analysis, unpaired t-test and cladogram from LEfSe analysis
